# Thirty-Day Unplanned Readmission After Ambulatory Laparoscopic Cholecystectomy in Western China: A Retrospective Study

**DOI:** 10.7759/cureus.13932

**Published:** 2021-03-16

**Authors:** Huang MingJun, Dai Yan, Li JiPing, Ma HongSheng

**Affiliations:** 1 Day Surgery Center, West China Hospital, Sichuan University, Chengdu, CHN; 2 West China School of Nursing, West China Hospital, Sichuan University, Chengdu, CHN; 3 Nursing Department, West China Hospital, Sichuan University, Chengdu, CHN

**Keywords:** ambulatory surgery, laparoscopic cholecystectomy, unplanned readmission

## Abstract

Background

Day surgery has been gradually accepted by health professions globally, which can shorten the hospital stay and reduce medical costs. The ambulatory laparoscopic cholecystectomy (LC) has been performed in China since over 10 years. However, few studies focus on its 30-day unplanned readmission rate of ambulatory LC and no standard of unplanned readmission rate for that now.

Purpose

This study aimed to evaluate the unplanned readmission rate and the reasons readmission after ambulatory LC in a tertiary hospital, which is the earliest ambulatory surgery implementation place in western China.

Methods

A retrospective analysis was conducted. The patients who underwent ambulatory LC from September 2015 to September 2019 in West China Hospital were screened. The 30-day unplanned readmission rate was calculated as the first outcome. The reasons for unplanned readmission were analyzed and classified as the second outcome.

Results

The study included 3,014 patients, and the unplanned readmission rate was 1.53%. The rate of patients diagnosed with cholecystolithiasis with cholecystitis was significantly higher in the unplanned readmission group (73.9% vs. 48.9%, p=0.003), and medical cost of unplanned readmission patients was significantly more than that of non-readmission patients (8,102.4±1,375.7 Yuan vs. 7,574.61±10,14.0 Yuan; p=0.008). It was observed that 71.7% readmission happened in the first seven days. Wound problems (60.9%) and abdominal pain (26.1%) went the two main reasons for unplanned readmission.

Conclusions

The analysis revealed that the unplanned readmission rate of 1.53% was low for ambulatory LC. Some causes of unplanned readmission, such as abdominal pain and wound site pain, wound exudate could be reduced by some simple interventions of the clinical professions.

## Introduction

Day surgery has been gradually accepted by health professions globally from its first establishment in the 20th century in the UK [1]. Day surgery is also called “ambulatory surgery” or “outpatient surgery” in different studies [2]. In fact, the classical definition of day surgery was announced in 2011 by the IAAS (International Association for Ambulatory Surgery): the patients must be admitted and discharged within a calendar day, with day surgery as an intended management [3]. Evidence has been found that day surgery can reduce the medical costs, shorten the hospital stay, and allow patients to recover at home [4]. From the report of Toftgaard and Parmentier [5], day surgery has taken over 90% of all elective surgery cases in the USA and Canada. Also, the percentage of day surgery in Denmark, Spain, and Sweden was 89%, 87%, and 80%, respectively [6]. The ambulatory laparoscopic cholecystectomy (LC) is one of the best clinical practice of day surgery. Since Philippe Mourret conducted the first case of LC in 1987 in France [7], the LC has spread with an incredible speed worldwide and has become the gold standard for the treatment and care for symptomatic gallbladder diseases [8] due to the similar outcomes of patients but shorter hospital stays compared to the traditional opening cholecystectomy [9]. The data showed that ambulatory LC presented in over 50% and 75% of all LC cases in some European countries and the USA, respectively [10].

Although the first day case surgery was performed in Hong Kong in the 1990s, Wuhan Children's Hospital performed the first case of day surgery until 2001 in mainland China [11]. In 2014, day surgery comprised approximately 35% of all selective surgery cases in China [12], and ambulatory LC comprised 26.7% of all LC cases [11]. Previous studies had found that the implementation of ambulatory LC decreased 22.3% of total medical costs, with no significant differences in complications rate and morbidity, compared to in-hospital LC [13]. Despite the cost saving, shorter hospital stay, and other advantages, the concerns about ambulatory LC, especially unplanned readmission, remain since there will be no cost benefits for patients undergoing day surgery if they go back to hospitalization for treatment again [14]. Therefore, unplanned readmission rate after discharge is a key indicator for the overall quality of care for day surgery [15]. Simsek et al. [7] analyzed 568 medical records of ambulatory LC cases in Turkey and found that the 30-day unplanned readmission rate was 2.4%. A multicenter retrospective analysis involving 230,745 ambulatory LC cases by Rosero and Joshi [14] revealed that the 30-day unplanned readmission was 2.02%. Moreover, a systematic review from Tang et al. [13] reported that the readmission rate of ambulatory LC was 2.4%. Although no standard readmission rate has been set up yet globally, previous studies believed that the unplanned readmission rate of over 2.0% was unacceptable. Thus, it is necessary to examine the unplanned readmission rate of ambulatory LC cases and its risk factors so that we can make progress in patient selection, operation procedure normalization, and post-operation care to reduce the unplanned readmission rate and improve the cost benefits of the ambulatory surgery. However, few studies have reported the 30-day unplanned readmission rate of ambulatory LC in China, where day surgery started just a few years. Therefore, this study aimed to evaluate the 30-day unplanned readmission rate and the reasons for readmission in patients receiving ambulatory LC.

## Materials and methods

Setting

A retrospective study was conducted in a day surgery unit of a tertiary medical center in west China. The study hospital launched ambulatory surgery in 2009 as the earliest ambulatory surgery implementation hospital in western China [16].

Data collection

Patients receiving ambulatory LC from September 2015 to September 2019 were screened. Data were extracted from the Health Information System and the Day Surgery Follow-up database of the hospital. The Health Information System included demographic data, day surgery procedure data, and other nonclinical information such as insurance type. The Day Surgery Follow-up database was established in 2009 to investigate the outcomes of patients on the second day, third day, and 30th day after discharging from the day surgery center. The follow-up staff inquired about patients’ status after discharged and recorded on the electronic follow-up system, and then instructed the patients about how to observe and deal with health status. This database consisted of a patient identifier (registered number in the Health Information System), date, reasons, diagnosis, and treatment for the unplanned readmission patients. The data were extracted from the separated database and combined through the registered number. The exclusion criteria were as follows: patients who had partial cholecystectomy, patients who had emergency LC, and patients who were readmitted without detailed reasons. The demographic data including age, gender, insurance type, and operation procedure were extracted directly from the Health Information System. The details of unplanned readmission, including the presenting complaints, days after discharge and treatment, were extracted from the Day Surgery Follow-up database directly.

Data analysis

Data were processed using SPSS Version 20.0 (IBM Corp., Armonk, NY, USA). Descriptive data were presented using the appropriate method: frequency and percentage for enumeration data and mean and standard deviation for consecutive variable data. The demographic, diagnosis, medical cost, and insurance type of patients were analyzed to compare differences between unplanned readmission patients and others. Continuous variables with equal variance were assessed using t-test or Mann-Whitney U test. Chi-square test or Fisher’s exact test was used for categorical variables, and test standard was α=0.05.

In this study, the unplanned readmission was defined as any use of emergency department, outpatient department, or other medical services during 30 days after the procedure, except prolonged hospital stay [17].

Ethic consideration

This study used the identified information (registered number) of patients to extract data and select participants. This study was approved by the Ethics Committee of West China Hospital of Sichuan University.

## Results

A total of 3,013 patients who underwent ambulatory LC were included in this study. Of all patients, 46 patients had unplanned readmission. The unplanned readmission rate at 30 days after the LC procedure was 1.53% in our study. Table 1 shows the detailed sociodemographic and clinical characteristics of the 46 patients and others. Most unplanned readmission patients were females (76.1%). The patients diagnosed with cholecystolithiasis accompanied by cholecystitis took the largest proportion of all unplanned readmission cases (73.9%).

**Table 1:** Sociodemographic and clinical characteristics of the readmission patients ASA, American Society of Anesthesiologists; URBMI, urban resident basic medical insurance; UEBMI, urban employee basic medical insurance

Characteristics	Readmission (N=46), mean (SD)/N (%)	Non-readmission (N=2967), mean (SD)/N (%)	t (95% CI)/χ²	p-Value
Age	38.5±10.7	40.6±10.6	1.270 (-1.11 to 5.18)	0.976
Gender				
Male	11 (23.9)	1,033 (34.8)	2.378	0.123
Female	35 (76.1)	1,934 (65.2)		
Marital status				
Married	40(87.0)	2,643 (89.1)	0.312	0.856
Unmarried	5(10.8)	252 (8.5)		
Divorced or widowed	1(2.2)	72 (2.4)		
Employment status				
In-service	44 (95.6)	2,918 (98.4)		
Retired	1 (2.2)	13 (0.4)	1.870	0.393
Unemployed	1 (2.2)	36 (1.2)		
ASA grade				
I	37 (80.4)	2,125 (71.6)	1.736	0.188
II	9 (19.6)	842 (28.4)		
Diagnosis				
Cholecystolithiasis	9 (19.6)	1,249 (42.1)	11.593	0.003
Cholecystolithiasis (with cholecystitis)	34 (73.9)	1,450 (48.9)		
Others	3 (6.5)	268 (9.0)		
Insurance type				
URBMI	5 (10.9)	169 (5.7)		
UEBMI	30 (65.2)	2,127 (71.7)	1.976	0.372
Out-of-pocket	11 (23.9)	671 (22.6)		
Medical costs	8,102.4±1,375.7 Yuan	7,574.61±1,014.0 Yuan	-3.172 Yuan (-854.04 to -201.50)	0.008

The medical cost of unplanned readmission patients was significantly higher than non-readmission patients (p<0.05), as shown in Table 1. The age, gender, marriage status, employment status, diagnosis, and insurance type had no significant difference between the two groups of patients (p>0.05).

Table 2 demonstrates the time and type of medical service for unplanned readmission. It was observed that 71.7% readmission happened in the first seven days. Most of the unplanned readmission patients called for medical help in the outpatient or emergency department (67.4%).

**Table 2:** Time and type of medical service for readmission (N=46)

Variables	N (%)
Days for readmission after the procedure	
1-7 days	33 (71.7)
8-14 days	9 (19.6)
15-30 days	4 (8.7)
Readmission type	
In-hospital	15 (32.6)
Outpatient/emergency department	31 (67.4)

Table 3 shows the diverse causes for unplanned readmission. The problems related to wound were the most common reason for unplanned readmission (60.9%). Of all wound problems, the wound exudate had the highest incidence rate (19.6%). During the first seven days after discharge, the top three reasons for unplanned readmission were abdominal pain (10/33), unhealed port/wound exudate (5/33), and wound infection (4/33). After the seventh day, the first cause for unplanned readmission was wound pain/exudate (4/13). One jaundice patient and one bile artery hemorrhage patient were readmitted to the hospital during the first seven days.

**Table 3:** Causes of readmission (N=46)

Readmission causes	N (%)
Urinary problems	4 (8.7)
Abdominal pain	12 (26.1)
Abdominal distention	2 (4.3)
Wound problems	28 (60.9)
Wound pain	7 (15.2)
Unhealed port	8 (17.4)
Wound infection	6 (13.0)
Wound exudate	9 (19.6)
Vomiting	2 (4.3)
Jaundice	1 (2.2)
Bile artery hemorrhage	1 (2.2)
Fever	2 (4.3)
Fatigue	1 (2.2)
Constipation	1 (2.2)

## Discussion

This study revealed that the 30-day unplanned readmission rate after ambulatory LC procedure was 1.53%. The rate of patients diagnosed cholecystolithiasis with cholecystitis were higher in unplanned readmission group than the non-readmission group (p<0.05), and the cost of it was also more than non-readmission group (p<0.05), but there was no difference in age, gender, marital status, employment status, ASA (American Society of Anesthesiologists) grade, and insurance type between two groups. Most of the unplanned readmissions happened in the first seven days after discharge. Furthermore, wound problems were the most common reason for patients’ medical problems. However, abdominal pain had the highest incidence rate during the first seven days.

There is no standard of unplanned readmission rate after ambulatory LC procedure, though the IAAS has been established for decades. It was surprising that the 30-day unplanned readmission rate was 1.53%, which is lower than the reported rate of 2.4% in Turkey [7]. A multicenter retrospective analysis involving 230,745 ambulatory LC cases conducted by Rosero and Joshi [14] revealed that the 30-day unplanned readmission rate was 2.0%. It was clear that the readmission rate was lower in our study compared to the previous two studies. First, we only included patients who underwent LC but excluded patients who underwent duct exploration, which was thought as a risk factor for unplanned readmission [14]. Second, we excluded patients who received emergency LC, which comprised 43% of all unplanned readmission cases in the study by Simsek et al. [7]. In one respect, the low readmission rate (1.53%) in our hospital could prove that China has the ability to carry out LC day surgery. Particularly, a previous study reported that the 30-day unplanned readmission rate was 5.9% in patients who received in-hospital LC [18]. Obviously, ambulatory LC had a lower incidence of 30-day unplanned readmission rate compared to the in-hospital LC. The reason leading to this discrepancy may be the strict inclusion and exclusion criteria in ambulatory surgery.

More patients in the readmission group were diagnosed with cholecystitis, and researchers indicated that complications in patients with cholecystitis were higher and that hospital stay were longer compared to symptomatic cholecystolithiasis [19]. It is reasonable that unplanned readmission would increase the medical cost for day surgery patients [20]. Other factors, such as population characteristics, did not differ in the readmission rate between the two groups. It indicated that the inclusion criteria for LC ambulatory surgery in our center were feasible; furthermore, the readmission rate of 1.53% may be unavoidable. If it was an acceptable incidence, the international standards for ambulatory surgery quality would have more reference.

It is found that the most unplanned readmission happened during the first seven days after discharge. Like opening cholecystectomy, ambulatory LC carries some postoperative complications as well, including wound pain, wound infection, vomiting, nausea, and urinary retention, at the early stage after surgery [21]. Rosero and Joshi [14] reported that 64.4% patients receiving ambulatory LC had unplanned readmission records in the first seven days after discharge, and 11.1% were readmitted in the first 24 hours after discharge. These findings were consistent with our result. Thus, it was important to provide enough information about the expected complications and the ways to cope with these complications at home [22]. Although pre-operation health education about the way to deal with excepted complications was a standard procedure in our hospital, 10 patients had unplanned readmission with complaints of abdominal pain in the first seven days in this study. Some factors contributing to unplanned readmission cannot be identified before the operation and simply avoided by patients at home [23], for example, persistent pain, vomiting, and nausea caused by anesthetic procedure were common factors that contributed to unplanned readmission but cannot be avoided and handled by patients. Hence, the use of appropriate anesthesia, analgesia, and anti-emetics in clinical practice may be crucial to relieve the severity of postoperative pain, vomiting, and nausea, leading to a decrease in unplanned readmission rate [24].

We also found that the wound problems, including pain, unhealed port, infection, and exudate, took the largest proportion of all unplanned readmission causes. In the hospital, we usually administer one dose of parecoxib (the COX-2 inhibitors) for analgesia before operation and another dose after surgery with a six-hour time interval. Additionally, three days’ medication of NSAIDs (nonsteroidal anti-inflammatory drugs) is prescribed after discharge. This may be not enough for some patients who are sensitive to post-operative pain. It was similar to the result from Rosero and Joshi [14], who found that 11.8% unplanned readmission patients had the complaint of wound pain. Rahimzadeh et al. [25] and Sharan et al. [26] injected ropivacaine or bupivacaine in the Trocar site to reduce post-operative wound pain. Hamad et al. [27] combined adrenaline and bupivacaine to inject in all ports to reduce the post-operative pain. Thus, these methods should be taken into consideration in clinical practice. Additionally, for patients who reported more severe pain before discharge, strengthened analgesic medication should be prescribed rather than the routine NSAID when they went home. It was not surprising to find that wound exudate and unhealed port became the top reasons for unplanned readmission, as the 5-mm ports are not sutured during the operation, which may increase the risk of unhealed port and wound exudate. To reduce the unplanned readmission rate through the decrease of unhealed port and wound exudate, it is important to give enough knowledge to patients about wound care at home. Moreover, some patients were readmitted because of wound infection. All over the world, it is a consensus not to use antibiotic after ambulatory LC unless there is definite evidence for intra-abdominal infection [28]. Furthermore, Uludag et al. [29] concluded that the prophylactic antibiotic cannot reduce the risk of wound infection. Under these conditions, some wound infection cases for unplanned readmission seems to be unavoidable, especially cholecystitis patients who underwent ambulatory LC procedure. More studies are needed to explore interventions for infection prevention.

It was not surprising to find that abdominal pain was one of the main causes of unplanned readmission. There are many factors contributing to abdominal pain after LC procedure, such as pain caused by carbon dioxide, incision, and complications [30]. It is important to identify the reason leading to pain, as abdominal pain, for example, may indicate some serious complications, including hemorrhage, bile leakage, and biloma, of LC procedure [7]. Simsek et al. [7] reported that abdominal pain was the first leading cause of unplanned readmission, of which 40% cases were finally diagnosed with biloma. Thus, if patients have an emergency/outpatient visit with the chief complaint of abdominal pain, do identify the specific reason. Unfortunately, in our study, we got the readmission rate but were unable to identify the reasons for abdominal pain by telephone follow-up, as some patients were readmitted to other hospitals where we cannot get information about the accurate diagnosis and treatment. In addition, only one jaundice case and one bile artery hemorrhage case were found in our study. These findings were in agreement with the previous result that ambulatory LC was associated with a lower incidence of bile artery hemorrhage (about 0.05%) [28]. This is also one of the reasons why LC can be conducted in the day surgery unit.

The major strength of this study is the large sample size. However, there are some limitations as well. First, this was a single-center data analysis. The different levels of medical services in different hospitals may lead to different unplanned readmission rate. Second, this study had no access to some records of the specific reason for unplanned readmission patients who seek medical services in other hospitals. This information is important to identify the specific reason for unplanned readmission.

## Conclusions

Unplanned readmission rate after discharge is a key indicator for the quality of ambulatory surgery care. The analysis reveals that the incidences of unplanned readmission rate and 30-day unplanned readmission of 1.53% are low for ambulatory LC. Investigating the incidence of unplanned readmission could help evaluate the standard for ambulatory surgery. This study clarified the reasons for unplanned readmission after discharge of patients who underwent ambulatory LC procedure. Wound problem and abdominal pain are important contributors to unplanned readmission. It was essential for clinical professions to improve education, strength analgesia management, and provide early assessment of complications before discharge to reduce the incidence of readmission.
